# It takes a village: an interview with Willias Masocha on the importance of networking in science

**DOI:** 10.1038/s42003-021-02157-3

**Published:** 2021-05-19

## Abstract

Willias Masocha is a Professor in the Department of Pharmacology & Therapeutics at Kuwait University where he studies the pathophysiology and treatment of neuropathic pain. Professor Masocha obtained a Bachelor of Pharmacy Honours from the University of Zimbabwe followed by a PhD in Pharmacology at the University of Granada before undertaking his postdoctoral training and International Brain Research Organization (IBRO) fellowship at the Karolinska Institutet. He began his independent research career at Kuwait University in 2006. In this Q&A he tells us about his current work and his perspectives on neuroscience research in the Middle East and Africa. He also shares tips for young scientists—particularly those based in Africa.

**Figure:**
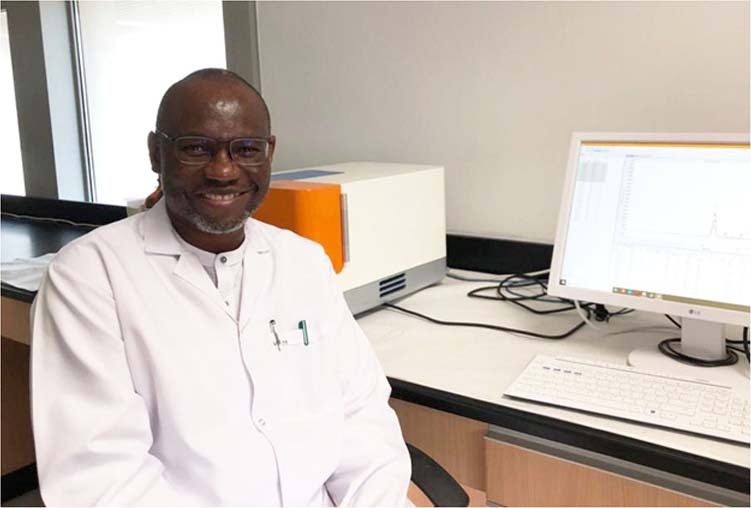
Esraa Aly

Can you tell us about your research interests?

My research focuses on studying the pathophysiology of pain and the evaluation of the activities of potential anti-inflammatory and/or analgesic drugs. I am also interested in studying neuroinflammation that occurs during neuropathic pain, as well as that induced by pathogen-related molecules such as lipopolysaccharide. However, of late, my focus has been on chemotherapy-induced neuropathic pain (CINP), antiretroviral drug-induced neuropathic pain or HIV-associated neuropathic pain (HIV-NP) and the endocannabinoid system. Chemotherapeutic drugs, such as paclitaxel and oxaliplatin, which are used for treating various cancers, and antiretroviral drugs that are used in the management of HIV, can cause neuropathy, which negatively impacts patient quality of life and might necessitate drug withdrawal. This therefore affects cancer or HIV treatment success. There is a dearth of drugs for proper management of both CINP and HIV-NP. Some studies suggested that cannabis can prevent the development of, or alleviate, established CINP and HIV-NP. However, the use of cannabis is associated with psychoactive effects. Part of my research is therefore aimed at evaluating the ability of non-psychoactive phytocannabinoids and plant extracts that modulate the endocannabinoid system, to prevent or alleviate CINP and HIV-NP in rodent models.

What motivated you to choose your career path?

I would say both serendipity and the need to contribute to the healthcare system. It has not been a straight path. Since childhood, I was fascinated with chemistry and medicine and so applied to do Pharmacy at University of Zimbabwe. During my undergraduate studies, I was fortunate to work as a research assistant in Professor Lameck Chagonda’s laboratory. The work involved extracting essential oils and doing chromatography to analyse their constituents. After graduation, I worked as a pre-registered pharmacist but I intended to work in the pharmaceutical industry and had secured a position at Datlabs, in Zimbabwe. However, before joining, I was informed by Prof Chagonda about a scholarship in Spain, which I applied for successfully.

I had intended to study natural products chemistry but I was aware of a colleague applying who had a Bachelors in Chemistry so I thought it would be better if I applied for Pharmacology given that we were supposed to be returning to the same department with our new skills. As a result, my intended goal shifted to the evaluation of compounds that were used against infectious pathogens. However, when I arrived at University of Granada I was advised to join Prof José Manuel Baeyens Cabrera’s laboratory as I did not speak Spanish at the time. As a result, my PhD focused on pain and evaluating the role of Na+, K+-ATPase on the antinociceptive effects of opioids. My interest in infectious diseases remained, however, so I took courses in Tropical medicine and Parasitology.

A pivotal time for my career was when I attended the first African IBRO school course in basic and clinical neuroscience in South Africa in 2000. There, I met Prof Marina Bentivoglio from the University of Verona who became my mentor. Part of her research is on sleeping sickness (African trypanosomiasis). She later connected me with Prof Krister Kristensson who had a research unit at Karolinska Institutet in Sweden. I did my post-doctoral training and IBRO research fellowship in Prof Kristensson’s lab focussing on the neurobiology of African trypanosomiasis, neuroinflammation and neuroimmunology. When I started my own lab at Kuwait University, I combined my experiences to focus on finding therapeutic agents to manage pain and neuroinflammation.

My research has gradually moved towards the endocannabinoid system for various reasons. Firstly, some of the drugs we have studied act in a cannabinoid receptor-dependent manner and secondly, some clinical trials show that smoked cannabis can alleviate HIV-NP. Lastly, but not least, since I was a teenager I have been fascinated with products of *Cannabis sativa* L. as therapeutic agents, more than as recreational substances, because although illegal at that time in Zimbabwe, it was used as part of traditional medicine for managing pain and other ailments such as asthma and epilepsy.

With roots in Africa and having lived in different parts of the world, how has your diverse personal background influenced you as a scientist?

Living in different parts of the world has helped me to be open, respectful of differences in approach to life and research, adaptable to various environments, and to value networking. I believe one can always do good research if they are determined despite any limitations, whether they are structural or financial. It is easier to form collaborations with others who might have different expertise or research equipment, if one has asked a good scientific question and performs their research with rigor utilising the resources available. For example, in 2013, I spoke at the European Workshop on Cannabinoid Research. One of the organisers found my talk interesting and we later agreed to collaborate because they had equipment and expertise that I did not have but was in need of.

I suppose also having roots in Africa has made me more interested in translational research and drug repurposing, since the latter can be cheaper, easier and faster to move from basic to clinical research. Some of the drugs we have evaluated in my lab in the context of neuropathic pain were already available in the market for managing other conditions such as epilepsy, general pain, inflammation and infections, or as food additives.

The other aspect that I value is sharing knowledge with young scientists and helping them to network. One activity that helped me immensely in my education and career was mentorship through IBRO Neuroscience schools. As I mentioned before, Prof Bentivoglio helped me define research questions that interested me and connected me with the contacts I needed to pursue those ideas. Over the years I have also become involved in organising, teaching, and mentoring young scientists in IBRO schools and the African School of Neuroimmunology (AfSNI). The latter is part of the Global Schools of Neuroimmunology (GSNI) of the International Society for Neuroimmunology (ISNI). Currently, we are working with over 50 neuroscientists from across the world to write articles that will contribute to an African Neuroimmunology Handbook for teaching at IBRO/ISNI schools in Africa. We hope that this also creates an interaction opportunity between IBRO and ISNI neuroscientists all over the world, including Africa.

What is the one quality you value over others when recruiting new group members?

When recruiting a new group member, instead of valuing one quality over others, I value a combination of qualities. I value scientific integrity, self-awareness, adaptability, enthusiasm and passion for science; a willingness to learn and putting skills into action, attention to details and being a team player. Some aspects such as good communication, technical expertise etc. are important but not at the top of my list because they can be easily taught or nurtured.

How do you see the neuroscience research landscape changing in the Middle East and Africa over the next decade?

Although lagging behind other parts of the world in terms of neuroscience education and research, I think there is now more awareness and a willingness to do neuroscience research in the Middle East and Africa. Over the next decade there will be more neuroscience education and research being done in this part of the world. Organizations such as IBRO have been investing a lot in schools to teach and nurture young neuroscientists. The establishment of IBRO African Centers for Advanced Training in Neuroscience has helped a lot in changing the landscape of neuroscience in Africa. Some universities such as the University of Cape Town have established Neuroscience Institutes, which is important for both educational and research purposes. The activities of neuroscience societies such as the Society of Neuroscientists of Africa (SONA), Mediterranean Neuroscience Society, Neuroscience Group in the United Arab Emirates, IBRO Middle East/North Africa Sub-Regional Committee (MENA), IBRO Africa Regional Committee (ARC), etc., are helping to move the neuroscience agenda forward in this part of the world.

What is your message to the next generation of scientists, particularly those based in Africa?

The two messages I would give to the next generation of scientists, particularly those based in Africa are: 1. Network with others, and 2. Do the best research you can with the resources available to you.

In addition to acquiring good education and research skills, it is essential to start networking early and find peers who share your passion for science and more importantly find good mentors. Establish long-term, meaningful scientific relationships and be ready to form more as you progress. Some of the easiest ways to network and know what is going on in the field is through attending webinars, workshops, and conferences. Identify scientific societies that you can become part of and actively participate in. There is a proverb that says “It takes a village to raise a child”. It will take a lot of members in the scientific community to nurture you into a good scientist and likewise, you should return the favour by nurturing other young scientists when you can.

Do the best research you can with the available resources even though they might sometimes be limited. Do not wait for a time when the environment is favourable or there are more resources available. Good science is demanding and needs perseverance, but it can also be fun and fulfilling. If you persevere in the long run, as your research question and findings take shape, you will either get more funding or be able to collaborate with others to take your research question to the next level. One of the famous scientists that my mentor used to refer to is Rita Levi-Montalcini, who was an Italian Nobel laureate, who did some research in a laboratory she set up at the cottage they were living in after moving from Turin because of the bombings during World War II. This example has always reminded me that with passion for science one can do meaningful research even under difficult circumstances.

*This interview was conducted by Associate Editor Karli Montague-Cardoso*.

